# A Glycolipid Adjuvant, 7DW8-5, Enhances the Protective Immune Response to the Current Split Influenza Vaccine in Mice

**DOI:** 10.3389/fmicb.2019.02157

**Published:** 2019-09-18

**Authors:** Huapeng Feng, Noriko Nakajima, Li Wu, Makoto Yamashita, Tiago J. S. Lopes, Moriya Tsuji, Hideki Hasegawa, Tokiko Watanabe, Yoshihiro Kawaoka

**Affiliations:** ^1^Division of Virology, Department of Microbiology and Immunology, The Institute of Medical Science, University of Tokyo, Tokyo, Japan; ^2^Department of Pathology, The National Institute of Infectious Diseases, Tokyo, Japan; ^3^Department of Pathobiological Sciences, School of Veterinary Medicine, University of Wisconsin-Madison, Madison, WI, United States; ^4^Aaron Diamond AIDS Research Center, Affiliate of the Rockefeller University, New York, NY, United States; ^5^Department of Special Pathogens, International Research Center for Infectious Diseases, Institute of Medical Science, University of Tokyo, Tokyo, Japan

**Keywords:** glycolipid, 7DW8-5, influenza vaccine, adjuvants, mice

## Abstract

Vaccination is an effective strategy to control influenza disease. Adjuvants enhance the efficacy of vaccines, but few adjuvants are approved for human use, so novel, safe, and effective adjuvants are urgently needed. The glycolipid adjuvant 7DW8-5 has shown adjuvanticity to malaria vaccine; however, its adjuvant effect for seasonal influenza vaccine remains unknown. Here, we evaluated the adjuvanticity of 7DW8-5 to a quadrivalent split influenza vaccine in a mouse model. 7DW8-5 significantly enhanced virus-specific antibody production when administrated with influenza vaccine compared with that of vaccine alone; 10 μg of 7DW8-5 induced similar antibody levels to those induced by alum. Mouse body weight loss was reduced and, notably, the survival rate was increased in the vaccine plus 7DW8-5 group compared with that in the vaccine plus alum group. Our results indicate that the glycolipid 7DW8-5 is a promising adjuvant for influenza vaccine.

## Introduction

Influenza virus causes seasonal influenza epidemics every winter and influenza pandemics every few decades, leading to serious economic and social disruption (Cauchemez et al., 2009; Gasparini et al., 2012). Annual vaccination is a useful means to control seasonal influenza (Houser and Subbarao, 2015; Rajao and Perez, 2018; Yamayoshi and Kawaoka, 2019). Currently, three types of licensed seasonal influenza vaccines are available: live attenuated, recombinant HA, and inactivated vaccines (Grohskopf et al., 2018). These vaccines are composed of three or four different types of influenza viruses (i.e., H1N1, H3N2, and one or two influenza B viruses), which are updated annually by the World Health Organization (WHO) to reflect the most recent circulating strains^1^. The inactivated influenza vaccines, including whole inactivated virus vaccine, split virus vaccine, and subunit vaccine, are widely used throughout the world; however, their efficacy is suboptimal, especially in the elderly^2^ (Wood and Siegrist, 2011; Choi et al., 2015; Dominguez et al., 2016; Flannery et al., 2018).

To improve vaccine efficacy, the addition of adjuvants is one of the most effective strategies. Adjuvants are substances that enhance the immune response to an antigen, and the ideal adjuvant would maximize vaccine efficacy and a strong safety profile (Petrovsky, 2015). Although several adjuvants are in clinical trials, many have failed to progress to approval for human use with vaccines due to toxicity, stability, biocompatibility, cost, and availability (Petrovsky and Aguilar, 2004; Sivakumar et al., 2011). Given that only a few adjuvants in combination with vaccines are currently approved for use in humans [e.g., alum, MF59, AS03, and AS04 (Mbow et al., 2010; Di Pasquale et al., 2015)], the development of novel and safe adjuvants is urgently needed.

The glycolipid alpha-Galactosylceramide (α-GalCer) binds CD1d, an MHC I-like molecule (Blumberg et al., 1991), which is primarily expressed by antigen-presenting cells (APC) and is presented to invariant natural killer T (*i*NKT) cells (Girardi and Zajonc, 2012). The adjuvant effect of α-GalCer has been investigated for vaccines against tumors and various infectious diseases, including influenza (Gonzalez-Aseguinolaza et al., 2002; Ko et al., 2005; Youn et al., 2007; Choi et al., 2008; Huang et al., 2008, 2013; Guillonneau et al., 2009; Kopecky-Bromberg et al., 2009; Kim et al., 2010; Miller et al., 2011; Lu et al., 2014; Artiaga et al., 2016). In fact, α-GalCer has been tested for several types of influenza vaccines (i.e., inactivated vaccines, live attenuated vaccines, and a DNA vaccine) in different animal models such as pigs and mice, and has been shown to enhance virus-specific antibody production and/or the protective efficacy of influenza vaccines, suggesting that α-GalCer is a promising adjuvant for influenza vaccines (Kamijuku et al., 2008; Guillonneau et al., 2009; Kopecky-Bromberg et al., 2009; Lee et al., 2011; Artiaga et al., 2016; Dwivedi et al., 2016; Fotouhi et al., 2017). Recently, Li et al. (2010) screened a library of 25 synthetic analog of α-GalCer, and identified a lead candidate, named 7DW8-5, that displayed a superior adjuvant effect for HIV and malaria vaccines in mice compared with the parental α-GalCer. For influenza vaccines, the adjuvanticity of 7DW8-5 has been tested for a DNA vaccine against H5N1 influenza virus (Hung et al., 2014); however, its adjuvant effects for other types of influenza vaccines are unknown.

In this study, we evaluated the adjuvant effect of 7DW8-5 on the commercial HA split vaccine in a mouse model. We found that 7DW8-5 enhanced the protective efficacy of the commercial HA split vaccine in mice, thereby demonstrating its promise as an adjuvant for the HA split influenza vaccine in humans.

## Materials and Methods

### Cells and Viruses

Madin-Darby canine kidney (MDCK) cells were maintained in minimum essential medium (MEM) (Gibco) supplemented with 5% newborn calf serum (Sigma) at 37°C in 5% CO_2_. MDCK cells were used for plaque assays to determine virus titers.

Mouse-adapted A/California/04/2009 virus (H1N1; MA-CA04), generated in our laboratory as previously described (Sakabe et al., 2011), was used to challenge mice. A/California/07/2009 virus (H1N1; CA07), which was isolated early in the 2009 pandemic, was used as an antigen for the ELISA after purification and inactivation to determine the virus-specific antibody titers of sera obtained from the immunized mice.

### Influenza Vaccine and Adjuvants

Quadrivalent split influenza HA vaccines were obtained from DENKA SEIKEN Co., Ltd. (Japan). The quadrivalent split influenza HA vaccine (for the 2016–2017 season), which contains the HA proteins (equivalent to 30 μg of HA protein for each virus included in a vaccine vial) of CA07 (H1N1), A/Hong Kong/4801/2014 (H3N2), B/Phuket/3073/2013 (Yamagata lineage), and B/Texas/2/2013 (Victoria lineage), was used. Aluminum hydroxide gel Alhydrogel^®^ adjuvant 2% (alum), purchased from InvivoGen, was used as a positive control [antigen: alum = 1:1 (v/v)] (approximately equal to 500 μg of alum/dose). The 7DW8-5 was purchased from Funakoshi Co., Ltd. (endotoxin level, <100 EU/mg), suspended in sterile water at a concentration of 1 mg/ml, heated at 80°C for 10 min, and then sonicated in a water bath for 15 min at room temperature. Stocks were stored at −20°C until use. Before being mixed with the split influenza HA vaccine, the 7DW8-5 suspension was sonicated again for 5 min after thawing. The 7DW8-5 and the HA split vaccine were diluted with the endotoxin-free D-PBS (Millipore, Cat. TMS-012-A, Endotoxin <0.005 EU/mL).

### Immunization and Protection

Five-week-old female BALB/c mice were purchased from Japan SLC Inc. After one week of adaptation, the mice (10 mice per group) were immunized with a suboptimal dose of influenza HA vaccine [0.001 μg/dose (2016–2017 season) calculated on the basis of the amount of HA from CA07] with or without 7DW8-5 into the gastrocnemius muscle. Two weeks later, the mice were boost immunized (second immunization) intramuscularly. On day 14 after the boost-immunization, blood was collected via the facial vein by using a goldenrod animal lancet (5 mm), and sera were obtained to measure virus-specific antibody titers. Three weeks after the boost-immunization, the immunized mice were challenged intranasally, under anesthesia, with 10 MLD_50_ (Fifty Percent Mouse Lethal Dose) of MA-CA04 virus. Body weight and survival of four mice from each group were monitored daily for 14 days after virus challenge. Mice that lost more than 25% of their original body weight were euthanized.

The remaining mice (6 mice per group) were used to assess virus replication. To determine virus titers in mice, organ samples were harvested on days 3 and 6 post-challenge and were homogenized and titrated on MDCK cells by using a plaque assay.

### Measurement of Virus-Specific Antibody Titers

Virus-specific antibody titers in the sera were determined by using a modified ELISA as previously described (Uraki et al., 2014). Briefly, 96-well ELISA plates (IWAKI) were coated with 6 μg/ml of inactivated, purified CA07 virus solution overnight at 4°C (50 μl/well). The plates were then blocked with 200 μl of 20% Blocking One (Nacalai) in water at room temperature for 1 h. After blocking, the plates were washed once with PBS containing 0.05% Tween-20 (PBS-T), and then 2-fold serially diluted serum samples were added to the plates, followed by a 1-h incubation at room temperature. Bound total IgG was detected by using peroxidase-labeled goat anti-mouse IgG (gamma) antibody, F (ab′) 2 fragment (Kirkegaard & Perry Laboratory Inc.) and horseradish peroxidase-conjugated anti-mouse IgG1, or IgG2a antibodies (Southern Biotech). After the plates were washed four times with PBS-T, 100 μl of 2, 2′-azino-bis (3-ethylbenzothiazoline-6-sulfonic acid) diammonium salt substrate solution was added to each well to initiate the color reaction, and the OD was measured at a wavelength of 405 nm. The antibody titer was defined as the reciprocal of the highest serum dilution that produced an OD_405_ > 0.1 after correcting for the negative serum control.

### Pathological Examination

Animal tissues were fixed in 4% paraformaldehyde phosphate (PFA) buffer solution for pathologic examination. They were then processed for paraffin embedding and cut into 3 μm-thick serial sections. The sections were stained using a standard hematoxylin and eosin procedure, and serial sections were processed for immunohistological staining with a rabbit polyclonal antibody for type A influenza virus nucleoprotein (prepared in the Department of Pathology, National Institute of Infectious Diseases, Tokyo, Japan) or a rat monoclonal antibody for mouse F4/80 antigen (CL: A3-1, Bio Rad). Specific antigen-antibody reactions were visualized with 3, 3′-Diaminobenzidine (DAB) staining by using a Dako Envision system (Dako Cytomation).

### Statistics

We used R^3^ and lme4 (Bates et al., 2015) to perform a linear mixed effects analysis of the body weight data, which were normalized to the initial weight of each individual animal. As fixed effects, we used the different treatment groups (i.e., vaccine alone, vaccine plus 7DW8-5 and vaccine plus alum), and the time of the measurement (with an interaction term between those fixed effects). As random effects, we used the intercepts for the individual animals. We used the lsmeans (Lenth, 2016) package to compare the groups at different time points for each model separately, and the *p*-values were adjusted using Holm’s method. For the comparisons of antibody titers and virus titers, we used a one-way ANOVA, followed by Tukey’s *Post Hoc* tests. The data were log-transformed before the comparisons were made to stabilize the variance. For the virus titer data, each timepoint was analyzed separately. For the antibody titers, values <10 were arbitrarily set to 5, because it was not possible to determine whether antibodies were completely absent or were present below the detection limit. For the analysis of the survival data, we used the Log-rank test, comparing the vaccine plus 7DW8-5 or alum to the vaccine alone group. We used OASIS 2 (Han et al., 2016) software for this analysis. *P* values of <0.05 were considered statistically significant.

### Ethics Statement

All experiments with mice were performed in the biosafety level 2 containment laboratory in the Institute of Medical Science, the University of Tokyo (Tokyo, Japan) in accordance with the Regulations for Animal Care of the University of Tokyo and the Guidelines for Proper Conduct of Animal Experiments by the Science Council of Japan, and were approved by the Animal Experiment Committee of the Institute of Medical Science, the University of Tokyo (approval no. PA 14-38).

## Results

### 7DW8-5 Significantly Enhances Influenza Virus-Specific Antibody Production in Mice

**FIGURE 1:**
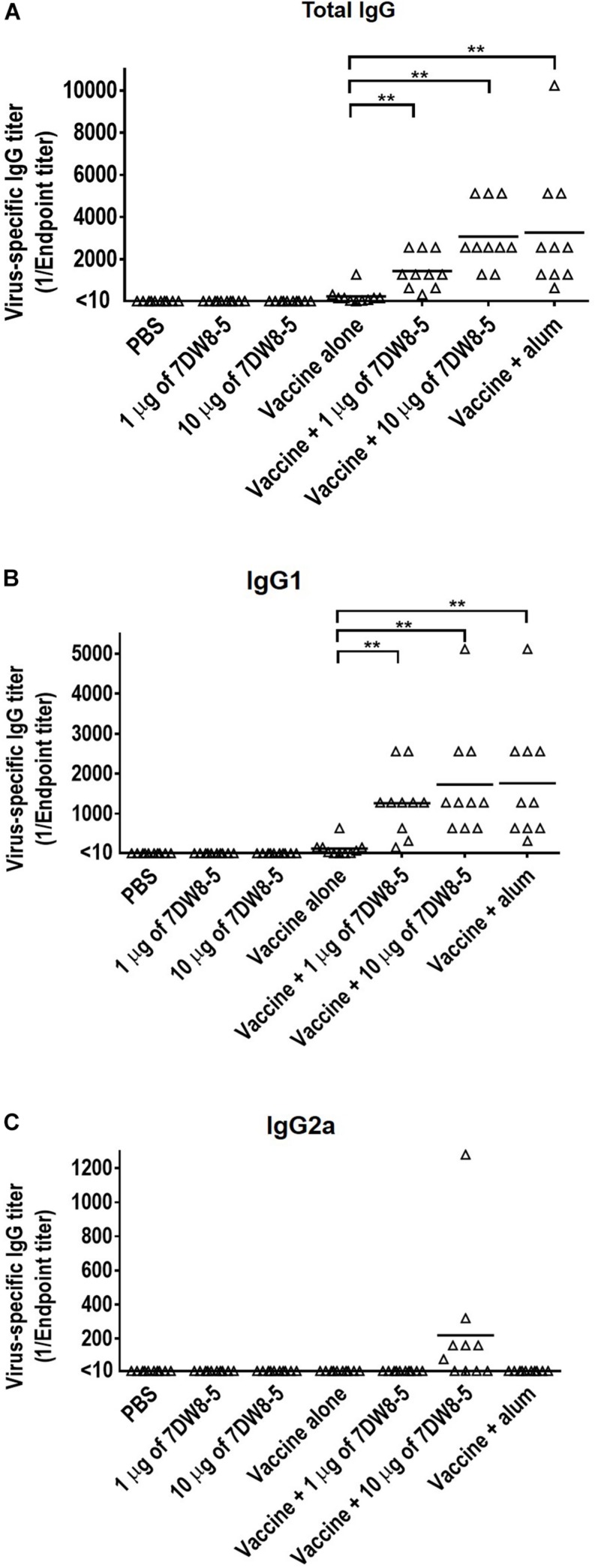
Virus-specific antibody titers induced by 7DW8-5 in combination with HA vaccine in mice. Six-week-old BALB/c mice (*n* = 10) were immunized with a commercial influenza HA vaccine with or without adjuvant twice with a 2-week interval between the vaccinations. Blood samples were collected 2 weeks after the second immunization. Virus-specific antibodies were measured by using an ELISA with inactivated and purified CA07 virus as the coating antigen. **(A)** Virus-specific total IgG antibody titers; **(B)** virus-specific IgG1 antibody titers; **(C)** virus-specific IgG2a antibody titers. The data were analyzed by using a one-way ANOVA followed by Tukey’s *Post Hoc* tests. The data were log2 transformed before the comparisons to stabilize the variance. The lines indicate the means of the antibody titers (*n* = 10). Values <10 were assigned the value 5 as described in the section “Materials and Methods.” ^∗∗^*P* < 0.01.

To evaluate the adjuvant effect of the glycolipid 7DW8-5 on a commercial HA split vaccine, mice were immunized with PBS, 7DW8-5 (1 μg/dose or 10 μg/dose), HA vaccine alone (0.001 μg of HA for each virus/dose), or HA vaccine plus 7DW8-5 (1 μg/dose or 10 μg/dose) via intramuscular administration in a 100 μl volume twice with a 2-week interval between the vaccinations. Commercially available alum adjuvant was used as a positive control, as described in the Materials and Methods, because alum is the most frequently used adjuvant worldwide and has been used in many clinical studies (Tetsutani and Ishii, 2012). Two weeks after the boost immunization, sera samples were obtained from the immunized mice and examined for the presence of virus-specific antibody in an ELISA. No antibody against CA07 virus was detected in the groups of mice that receive PBS only or 7DW8-5 only. Most mice immunized with the HA vaccine alone produced no or very low levels of virus-specific antibodies except for one mouse whose virus-specific IgG titer was 1280 (Figure 1). The mean antibody titer in the sera of the group of mice immunized with the HA vaccine plus 1 μg of 7DW8-5 (i.e., 1440.0) was significantly higher than that in the vaccine alone group (i.e., 240.5) (Figure 1). The vaccine plus 10 μg of 7DW8-5 also induced much higher levels of virus-specific antibody compared with the vaccine alone group, and the mean antibody titer was comparable to that induced by HA vaccine plus alum (Figure 1). These results demonstrate that 7DW8-5 significantly enhances the immunogenicity of the HA split vaccine.

We also measured the influenza virus-specific IgG1 and IgG2a titers in the sera by using an ELISA because IgG1 and IgG2a are stimulated during Th2-type and Th1-type immune responses, respectively (Stevens et al., 1988; Mosmann and Coffman, 1989; Hauge et al., 2007). The IgG1 titers in the HA vaccine plus 1 μg of 7DW8-5, the HA vaccine plus 10 μg of 7DW8-5, and the HA vaccine plus alum groups were significantly higher than that in the HA vaccine alone group (Figure 1B). Seven of the ten mice immunized with the HA vaccine plus 10 μg of 7DW8-5 expressed a high level of IgG2a antibodies, whereas no IgG2a antibody was detected in the other groups (Figure 1C). These results suggest that the HA vaccine plus 10 μg of 7DW8-5 induced both Th1-type and Th2-type immune responses, whereas the HA vaccine plus 1 μg of 7DW8-5 and the HA vaccine plus alum induced only the Th2-type immune response.

### 7DW8-5 Enhances the Protective Efficacy of Influenza Vaccine Against Lethal Virus Challenge in Mice

**FIGURE 2:**
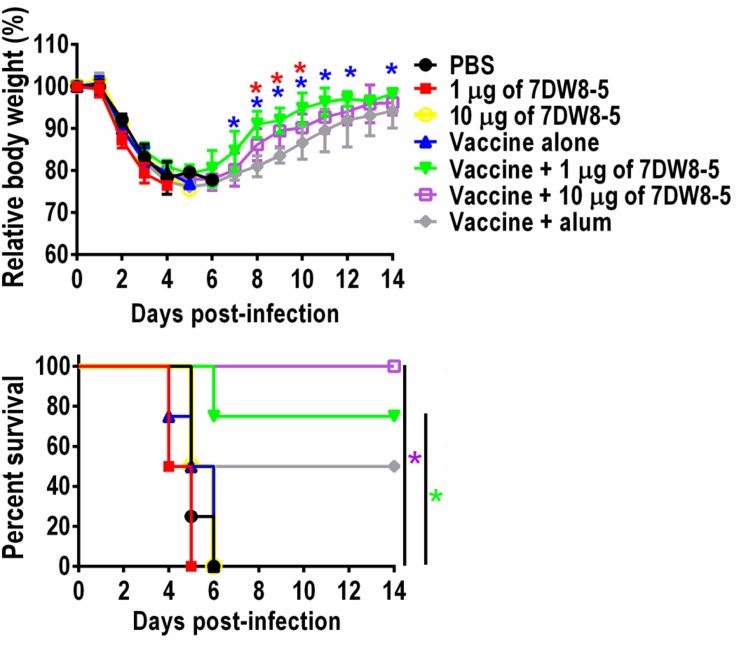
Protective efficacy of 7DW8-5 plus vaccine against lethal challenge. Six-week-old BALB/c mice (*n* = 4) were mock-immunized with PBS or 7DW8-5 alone, immunized with HA vaccine alone or 7DW8-5-adjuvanted HA vaccine twice with a 2-week interval between vaccinations. Mice were intranasally challenged with 10 MLD_50_ of MA-CA04 virus 3 weeks after the second immunization. Body weight and survival were monitored daily for 14 days. The body weight data shown are means ± standard deviation (SD). Green asterisks indicate a significant difference between the vaccine alone group and the vaccine plus 1 μg of 7DW8-5 group; purple asterisks indicate a significant difference between the vaccine alone group and the vaccine plus 10 μg of 7DW8-5 group; blue asterisks indicate a significant difference between the vaccine plus 1 μg of 7DW8-5 group and the vaccine plus alum group; red asterisks indicate a significant difference between the vaccine plus 10 μg of 7DW8-5 group and the vaccine plus alum group. ^∗^*P* < 0.05.

To examine whether 7DW8-5 enhances the protective efficacy of the HA split vaccine, we challenged the immunized mice with 10 MLD_50_ of MA-CA04 virus 3 weeks after the boost-immunization and monitored their body weight changes and survival for 14 days. All mice given PBS or 7DW8-5 alone experienced body weight loss upon virus challenge and died by 6 days post-challenge, whereas 2 of 4 mice were protected from lethal challenge in the vaccine plus alum group (Figure 2 and Supplementary Table 1). More importantly, 3 of 4 mice immunized with the vaccine plus 1 μg of 7DW8-5 survived and all four mice that received the vaccine plus 10 μg of 7DW8-5 were protected from the lethal infection (Figure 2 and Supplementary Table 1). These results demonstrate that the adjuvanticity of the glycolipid adjuvant 7DW8-5 was sufficient to protect mice from lethal challenge with MA-CA04 virus.

### HA Vaccine Plus 7DW8-5 Did Not Completely Prevent Challenge Virus Replication in Immunized Mice

To examine the effect of 7DW8-5 on virus replication in the immunized mice after challenge, the mice immunized with HA vaccine plus 7DW8-5 were challenged with 10 MLD_50_ of MA-CA04 virus 3 weeks after the second immunization, and organ samples (i.e., nasal turbinates and lungs) were collected from the sacrificed mice on days 3 and 6 post-infection for virus titration. On day 3 post-challenge, over 10^6^ PFU of virus was detected in both the nasal turbinates and lungs of mice in all of the groups (Table 1). In contrast, on day 6 post-challenge, the mean virus titers in the lungs for the vaccine plus 7DW8-5 and the vaccine plus alum groups were lower than that of the vaccine alone group, although the difference was not statistically significant (Table 1).

**TABLE 1:** Virus replication in the respiratory tract of immunized mice challenged with MA-CA04 virus^a^.

**Immunogen**	**Mean virus titers (Log _10_ PFU/g) ± SD**			
	**NT**		**Lungs**	
	**Day 3 p. i.**	**Day 6 p. i.**	**Day 3 p. i.**	**Day 6 p. i.**
PBS	6.3 ± 0.0	5.0 ± 0.6	7.3 ± 0.1	5.6 ± 0.3
1 μg 7DW8-5	6.5 ± 0.4	5.6 ± 0.5	7.2 ± 0.1	6.2 ± 0.4
10 μg 7DW8-5	6.9 ± 0.4	5.8 ± 0.1	7.3 ± 0.0	6.6 ± 0.1
Vaccine alone	6.0 ± 0.0	4.7 ± 0.5	7.2 ± 0.1	6.0 ± 0.4
Vaccine + 1 μg 7DW8-5	6.2 ± 0.1	4.2 ± 1.2	7.1 ± 0.1	5.3 ± 0.4
Vaccine + 10 μg 7DW8-5	6.1 ± 0.0	5.0 ± 0.2	7.0 ± 0.1	4.6 ± 1.7
Vaccine + Alum	6.2 ± 0.1	5.0 ± 0.4	7.2 ± 0.0	4.0 ± 1.3

*^*a*^Six-week-old BALB/c mice were immunized with the indicated immunogens (100 μl) twice (2-weeks apart) and challenged with 10 MLD_50_ of MA-CA04 virus 3 weeks after the second immunization. The nasal turbinates (NT) and lungs were collected from the mice (*n* = *3*) on days 3 and 6 post-infection (p. i.) and viral titers were determined in MDCK cells by use of plaque assays.*

**FIGURE 3:**
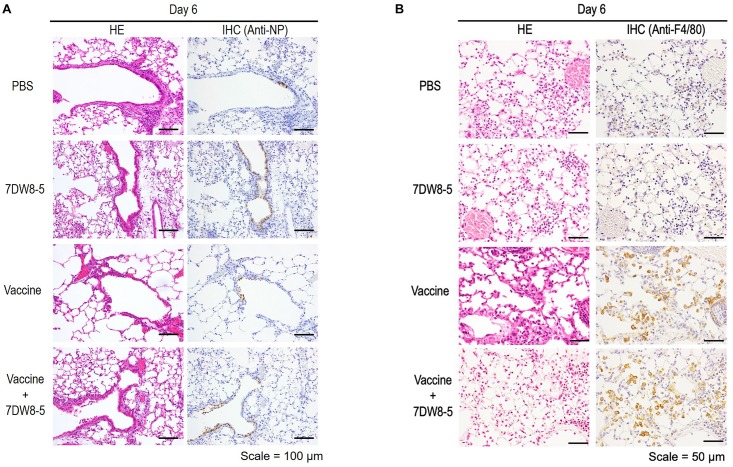
Pathological analysis of the lungs of immunized mice after challenge. Six-week-old BALB/c mice (*n* = 3) were immunized with PBS, 7DW8-5 (10 μg/dose), HA vaccine alone, or HA vaccine plus 7DW8-5 (10 μg/dose) twice with a 2-week interval between vaccinations. The immunized mice were intranasally challenged with 10 MLD_50_ of MA-CA04 virus 3 weeks after the second immunization. The lungs were fixed with 4% PFA buffer solution on day 6, and stained as described in the Materials and Methods section. HE, hematoxylin and eosin staining; IHC, immunohistochemistry for the detection of influenza virus NP antigen **(A)** or anti-F4/80 antibody **(B)**. Scale bars: 100 μm **(A)** and 50 μm **(B)**.

Pathological analysis of mice immunized with PBS, 7DW8-5 (10 μg/dose), HA vaccine alone (0.001 μg/dose), or HA vaccine plus 7DW8-5 (10 μg/dose) after challenge with 10 MLD_50_ MA-CA04 virus revealed that infection with MA-CA04 resulted in detectable viral antigens in bronchial epithelial cells and in infiltration of inflammatory cells into the lungs of all mice tested on day 6 post-challenge (Figure 3A). No apparent difference in inflammation, viral antigen distribution pattern, or the number of viral antigen-positive cells was observed across all groups (Figure 3A). Moderate inflammation, including infiltration of neutrophils, monocytes/macrophages, or lymphocytes, was observed in more than half of the sections from each mouse across all groups; however, we detected more F4/80-positive cells in the lungs of immunized mice after virus challenge in the HA vaccine alone and the 7DW8-5 plus HA vaccine groups compared with the PBS and 7DW8-5 groups (Figure 3B). These results suggest that immunization of mice with the HA vaccine induces infiltration of macrophages into the lungs after virus infection, although no difference in the number of F4/80-positive cells was observed between the HA vaccine alone and the 7DW8-5 plus HA vaccine groups.

Taken together, our results demonstrate that although 7DW8-5 did not facilitate a reduction in virus replication by the HA vaccine, it did improve vaccine efficacy as evaluated by lethality.

## Discussion

The use of adjuvants is an effective approach to improve vaccine efficacy. The CD1d-binding glycolipid α-GalCer is recognized as a member of a new class of adjuvants and its adjuvanticity has been investigated for vaccines against tumors and various infectious diseases, including influenza (Gonzalez-Aseguinolaza et al., 2002; Ko et al., 2005; Youn et al., 2007; Choi et al., 2008; Huang et al., 2008, 2013; Guillonneau et al., 2009; Kopecky-Bromberg et al., 2009; Kim et al., 2010; Miller et al., 2011; Lu et al., 2014; Artiaga et al., 2016). In this study, we evaluated the adjuvanticity of the glycolipid 7DW8-5, a novel analog of α-GalCer that showed a superior adjuvant effect compared with that of α-GalCer in malaria and HIV vaccines (Li et al., 2010), for a commercial influenza HA split vaccine in a mouse model. Although the adjuvant effect of 7DW8-5 on the DNA vaccine for H5N1 influenza virus was less than that of the parental compound α-GalCer (Hung et al., 2014), 7DW8-5 did enhance virus-specific antibody production and the protective efficacy of a commercial HA split vaccine against a lethal challenge of influenza A virus in mice. The safety and adjuvanticity of α-GalCer have been demonstrated in humans in Phase I clinical trials (Giaccone et al., 2002; Ishikawa et al., 2005; Nicol et al., 2011). In addition, 7DW8-5 has been shown to have a good safety profile and potent immune-enhancing activity in a non-human primate model (Padte et al., 2013). Additionally, we found that 7DW8-5 has a dose-sparing effect on the HA vaccine because the mean titer of the virus-specific antibody in mice immunized with 0.03 μg of HA vaccine alone was 2560, as described in our previous study (Feng et al., 2019), which is comparable to that in mice immunized with 0.001 μg of HA vaccine plus 10 μg of 7DW8-5 (mean antibody titer: 3072) in this study. These findings, coupled with our results, suggests that the glycolipid 7DW8-5 could be a promising adjuvant for the commercial HA split influenza vaccine.

Previous studies have shown that the induction of IgG2a antibodies, which occurs during the Th1-type immune response, is associated with increased efficacy of influenza vaccines (Huber et al., 2001, 2006; Proietti et al., 2002; Hovden et al., 2005). In contrast, inactivated influenza vaccines and subunit vaccines induce the Th2-type immune response, which is associated with the stimulation of IgG1 antibodies in BALB/c mice (Hocart et al., 1989; Benne et al., 1997; Moran et al., 1999). In the current study, we found that mice immunized with the HA vaccine plus 10 μg of 7DW8-5 had increased titers of both IgG1 and IgG2a antibodies (Figure 1C), suggesting that both Th1-type and Th2-type immune responses were induced by vaccination with the HA vaccine plus 10 μg of 7DW8-5. Additionally, pathological analysis showed that immunization of mice with the HA vaccine or with 7DW8-5 plus the HA vaccine induced macrophage infiltration into the lungs after virus challenge (Figure 3B). The induction of both Th1- and Th2-type immune responses in addition to macrophage infiltration may have facilitated viral clearance from the lungs of the infected mice immunized with the HA vaccine plus 10 μg of 7DW8-5, resulting in 100% protection (Figure 2).

The current influenza vaccines induce strain-specific antibody responses against viral HA proteins; however, strain-specific antibodies can only provide a reasonable measure of protection if the vaccine strains match the antigenicity of the current circulating strains (Pica and Palese, 2013; Khurana, 2018). Therefore, an approach aimed at broadening the cross-reactivity of influenza vaccines by targeting T cell responses is of considerable interest. The glycolipid α-GalCer activates *i*NKT cells, leading to the production of Th1 and Th2 cytokines (Bendelac et al., 2007), and the subsequent stimulation of various immune cells including dendritic cells (Fujii et al., 2003, 2006), natural killer (NK) cells (Kawano et al., 1997; Brossay et al., 1998), B cells (Kitamura et al., 2000), and CD4^+^ and CD8^+^ T cells (Singh et al., 1999; Hermans et al., 2003). Guillonneau et al. showed that α-GalCer enhanced T cell-mediated immune responses in mice immunized with an inactivated influenza virus vaccine and protected the immunized mice from heterologous influenza A virus challenge (Guillonneau et al., 2009). Therefore, the use of α-GalCer, and its analogs such as 7DW8-5, as an adjuvant could be an effective strategy to compel vaccines to elicit broader immune responses.

## Data Availability

The datasets generated for this study are available on request to the corresponding author.

## Ethics Statement

All experiments with mice were performed in the biosafety level 2 containment laboratory in the Institute of Medical Science, the University of Tokyo (Tokyo, Japan) in accordance with the Regulations for Animal Care of the University of Tokyo and the Guidelines for Proper Conduct of Animal Experiments by the Science Council of Japan, and were approved by the Animal Experiment Committee of the Institute of Medical Science, the University of Tokyo (approval no. PA 14-38).

## Author Contributions

HF, MY, TW, and YK designed the experiments. HF and LW performed most of the experiments. NN and HH performed the pathological analysis. MT provided material for the pilot experiments. HF, TL, TW, and YK analyzed the data. TW and YK oversaw the study. HF, TW, and YK wrote the manuscript. All authors reviewed and approved the manuscript.

## Conflict of Interest Statement

YK has received speaker’s honoraria from Toyama Chemical and Astellas and grant support from Chugai Pharmaceuticals, Daiichi Sankyo Pharmaceutical, Toyama Chemical, Tauns Laboratories, Otsuka Pharmaceutical, and Kyoritsu Seiyaku; and is a founder of FluGen. The remaining authors declare that the research was conducted in the absence of any commercial or financial relationships that could be construed as a potential conflict of interest.

## Footnotes

1 https://www.who.int/influenza/vaccines/virus/recommendations/en/

2 https://www.cdc.gov/flu/professionals/vaccination/effectiveness-studies.htm

3 www.r-project.org
